# *In vivo* antiaging effects of alkaline water supplementation

**DOI:** 10.1080/14756366.2020.1733547

**Published:** 2020-02-27

**Authors:** Mariantonia Logozzi, Davide Mizzoni, Rossella Di Raimo, Mauro Andreotti, Daniele Macchia, Massimo Spada, Stefano Fais

**Affiliations:** a Department of Oncology and Molecular Medicine, Istituto Superiore di Sanità, Rome, Italy; b National Center for Global Health, Istituto Superiore di Sanità, Rome, Italy; c Center of Animal research and Welfare, Istituto Superiore di Sanità, Rome, Italy

**Keywords:** Alkaline water supplementation, antiaging, telomerase, telomeres length, antioxidant effect

## Abstract

Telomeres length and telomerase activity are currently considered aging molecular stigmata. Water is a major requirement for our body and water should be alkaline. Recent reports have shown that aging is related to a reduced water intake. We wanted to investigate the effect of the daily intake of alkaline water on the molecular hallmark of aging and the anti-oxidant response. We watered a mouse model of aging with or without alkaline supplementation. After 10 months, we obtained the blood, the bone marrow and the ovaries from both groups. In the blood, we measured the levels of ROS, SOD-1, GSH, and the telomerase activity and analysed the bone marrow and the ovaries for the telomeres length. We found reduced ROS levels and increased SOD-1, GSH, telomerase activity and telomeres length in alkaline supplemented mice. We show here that watering by using alkaline water supplementation highly improves aging at the molecular level.

## Introduction

A progressive and apparently unstoppable process of becoming older day-by-day, also called aging, is indeed a physiological process common to almost all living beings, but Prokaryotes, Protozoans and algae that are innately immortal^1^^,^^2^. Previous literature strongly supports a conceivable multifactorial pathogenesis of aging including pre-determined and damage-related factors^3^. Moreover, a broad spectrum of diseases has been related to aging including tumours and neurodegenerative diseases^4–8^.

From a molecular point of view, the structures that are directly involved in the cellular aging are telomeres^9^^,^^10^. Telomeres are regions of highly repeated DNA at each end of chromosomes, which protect these regions from recombination, degradation and inter-chromosomal fusion^5^^,^^6^^,^^11^. Mammalian telomeres are composed of tandem repeats of the TTAGGG sequences associated with six telomere-specific sheltering protein complex that protects chromosome ends^8^^,^^11–13^. Thus, all investigations aimed at investigating aging from a molecular point of view have measured both the telomerase (the enzyme directly involved in telomeres elongation) activity and the telomeres length^5^^,^^6^^,^^14^.

However, while it appears clear to date that telomeres shortening is a molecular evidence of a progressive aging at the cellular level, there is poor information on which mechanism/s may lead to this apparently unstoppable process^3^^,^^15^. Some recent evidence suggests first that the progressive accumulation of toxics, such as oxidants, in our body may quicken aging at the cellular level^16–18^. Thus, one possible way to face off with aging is to reduce oxidants production and subsequent accumulation into tissues. This of course appears highly conceivable and reasonable; however, we do not have so many arrows to our bow in order to try a feasible anti-oxidant strategy^19^.

One of the most important requirements for our body is water. Water is one of the essential requirements for life that our body does not produce (i.e. there are not genes codifying for water production)^20^^,^^21^. On the other hand, our body is resilient enough to work even with low water intake. It is a matter of fact that, independently from the gender, elderly people use to have around the half of the body water % as compared to the new-borns, and this is probably due to the fact that they drink less water^21^^,^^22^.

Moreover, a recent study has reported that telomeres length may be highly influenced by microenvironmental pH variation^23^. In fact, human telomerase can be induced to selectively extend short telomeres by exposure to low pH (6.8)^23^. It is well known in fact that malignant tumours microenvironment is acidic and alkalinisation of tumour microenvironment highly impair tumour growth and progression^24^^,^^25^. However, tissue acidification is a metabolic hallmark of type II diabetes as well^26^. Of interest shorter telomeres, but not average telomere length, are related to reduced cell viability and senescence^27^. In fact, telomeres shortening triggers a DNA damage response, inevitably leading to cell apoptosis or senescence^8^^,^^27^. Low levels of telomerase, e.g. due to extracellular low pH, is related to short telomeres, a critical condition rapidly ending with cell death and senescence^23^. While a critical role of telomeres in cancer and aging appears clear, the mechanism that *in vivo* may influence telomeres either shortening or lengthening are not sufficiently explored^4–6^^,^^9^^,^^10^^,^^28–32^. One hypothesis suggests that telomere lengthening is regulated by preferential recruitment of telomerase to short telomeres; one other hypothesis suggests that telomerase exists in an inactive or active state, and that it is inactive on long telomeres and active on short telomeres^33^. Both these hypotheses do not appear mutually exclusive, and most of all we do not know whether one may be more operative in normal aging and the other in favouring diseases like cancer.

In a previous investigation, we have shown that oral treatment with Fermented Papaya (FPP^®^) induces clear tumour growth inhibition in a model of a very aggressive melanoma; this result was consistent with a decrease in reactive oxygen species (ROS) and an increase of natural anti-oxidant molecules such as SOD-1 and glutathione in the blood of treated mice^34^. However, it has been reported that *in vivo* administration of alkalinised water induced a delay in the aging of mice^35^, supporting a key role of alkaline water in at least slowing down the aging process.

Moreover, there are some molecules that have shown to be key in many aging-related processes (i.e. boric acid, potassium chloride, sodium chloride, potassium hydroxide, sodium molybdate dihydrate, sodium selenite). In particular, boron, molybdenum and selenium exert very important functions in the human metabolism processes. Boron is an essential element in many metabolic pathways of humans and animals^36^. Furthermore, boron is involved in the induction of an antioxidant response against ROS^36–38^, in the calcium metabolism and consequently in the bone growth^39^, with a potential role in the bone loss of the postmenopausal period^40^. Molybdenum is an essential trace element in most organisms with a key role in life expectance^41^. Molybdenum-containing enzymes, using water as oxygen donor or acceptor, catalyse the oxidation and sometimes reduction of small molecules in the metabolism of nitrogen, sulphur and carbon^42^. Deficiencies in molybdenum are associated with oesophageal cancer^43–45^, with high levels of sulphite and urate and with neurological damage^46^^,^^47^. Selenium is an essential trace element naturally contained in many foods including fish, meat, milk and derivatives, brewer’s yeast, cereals, nuts, mushrooms, fruits and vegetables^48^. Selenium is an essential requirement for a broad spectrum of metabolic activities, thanks to its antioxidant activity^49^. Metabolic diseases such as hyperglycaemia, hyperlipidaemia, and hyperphenylalaninemia are characterised by selenium deficiency, while a supplementation of selenium is associated with beneficial effects in atherosclerosis, hypercholesterolaemia, type 1 diabetes mellitus and phenylketonuria^49^.

The aim of this study was to investigate a potential role of alkaline water supplemented with the above-discussed elements in improving the molecular signature of aging, such as telomerase activity and telomeres length, consistent with the induction of a systemic antioxidant response.

## Materials and methods

### Alkaline water supplementation

Alkaline water supplementation (AWS) was obtained adding five drops of AlkaWater^®^ in 250 ml of tap water, until pH 9.0.

AlkaWater^®^ consists of boric acid, distilled water, potassium chloride, sodium chloride, potassium hydroxide, sodium molybdate dihydrate and sodium selenite.

AWS was administered orally by mice drinker at volume of 250 ml/kg/mouse (5 ml/mouse) every day without interruption, corresponding to concentration of 215 mg/kg/mouse/day (3.6 µl/mouse/day) of AlkaWater^®^. The daily treatment of the mice with the AWS started 2 weeks after the mice got to the animal facility (6 weeks of age) and continued for 10 months until the sacrifice of the mice (51 weeks of age).

The daily intake of each supplemented element through AlkaWater’s is shown in Table 1. The salts composition of AlkaWater^®^ is shown in Table 2.

**Table 1.** Concentration of daily AlkaWater’s elements consumption from mice.

Elements	Source	Concentration in 250 ml H_2_O (µg)	Daily consumption concentration from mice (µg element/mouse/day)
Boron	Boric acid	300	6
Molybdenum	Sodium molibdate [molybdenum (VI)]	10	0.2
Selenium	Sodium selenite	15	0.3

**Table 2.** Salts present in AlkaWater^®^.

Salt	Source	%
Sodium Chloride (NaCl)	Bolivian Rose™—Andes Mountain Salt from Andes Mountain Range (Bolivia) (Woodinville, WA, USA)	95.5
	Natural Alps Mountains Salt, coarse from Bergkern (Austria)	94.5
	HALITE SALT from rock salt mines (Pakistan)	99.35
Sodium (Na)	Bolivian Rose™—Andes Mountain Salt from Andes Mountain Range (Bolivia) (Woodinville, WA, USA)	–
	Natural Alps Mountains Salt, coarse from Bergkern (Austria)	35.4
	HALITE SALT from rock salt mines (Pakistan)	–
Calcium (Ca)	Bolivian Rose™—Andes Mountain Salt from Andes Mountain Range (Bolivia) (Woodinville, WA, USA)	0.7
	Natural Alps Mountains Salt, coarse from Bergkern (Austria)	0.25
	HALITE SALT from rock salt mines (Pakistan)	0.19
Magnesium (Ma)	Bolivian Rose™—Andes Mountain Salt from Andes Mountain Range (Bolivia) (Woodinville, WA, USA)	0.208
	Natural Alps Mountains Salt, coarse from Bergkern (Austria)	0.1
	HALITE SALT from rock salt mines (Pakistan)	0.16
Potassium (K)	Bolivian Rose™—Andes Mountain Salt from Andes Mountain Range (Bolivia) (Woodinville, WA, USA)	0.646
	Natural Alps Mountains Salt, coarse from Bergkern (Austria)	0.3
	HALITE SALT from rock salt mines (Pakistan)	–
Iron (Fe)	Bolivian Rose™—Andes Mountain Salt from Andes Mountain Range (Bolivia) (Woodinville, WA, USA)	0.303
	Natural Alps Mountains Salt, coarse from Bergkern (Austria)	–
	HALITE SALT from rock salt mines (Pakistan)	–
Sulphate (SO4)	Bolivian Rose™—Andes Mountain Salt from Andes Mountain Range (Bolivia) (Woodinville, WA, USA)	–
	Natural Alps Mountains Salt, coarse from Bergkern (Austria)	2.0
	HALITE SALT from rock salt mines (Pakistan)	0.13

### In vivo studies

All the studies were approved by the ethical committee of the Italian National Institute of Health (Rome) and were conducted in accordance with the current Italian Law (Law 26/2014), authorisation n. 792/2017-PR, that regulates experiments in laboratory animals. 20 C57BL/6J female mice between 16 and 20 g (4 weeks of age) were purchased from Charles River Laboratories Italia s.r.l., (Calco, Lecco, Italy), and housed in the animal facility of the Italian National Institute of Health. Mice had 10 and14 h periods of light and darkness respectively, were housed in a different number of animal cages, depending on the experiment, with *ad libitum* mice chow [Mucedola, Settimo Milanese (MI), Italy] and water provided through a bottle. Mice were checked twice a week by a veterinarian responsible for animal welfare monitoring for signs of sufferance such as weight loss, decreased water and food consumption, poor hair coat, decreased activity levels accordingly to the guidelines for a correct laboratory practice and signs of poor quality of life. Endpoint was 11 months old to evaluate aging parameters. Mice were divided into two groups: a control group (CTR group, untreated group) and a treatment group (AWS group). Each group consisted of 10 animals for statistical significance. CTR group received tap water (pH = 7) every day while AWS group was treated with AWS (pH = 9.0) every day without interruption until animal sacrifice. Each treated mice drank 5 ml of AWS every day, corresponding to 3.6 µl/mouse/day of AlkaWater^®^. Just before mice sacrifice, blood was withdrawn from mice eyes. Immediately after the sacrifice, bone marrow was isolated from both tibias and femurs of the mice hind legs, while ovaries were retrieval from reproductive system. Blood, bone marrow cells and ovarian germ cells were used for subsequent experimental analysis of aging parameters.

### Experimental study design

**Figure 1.**
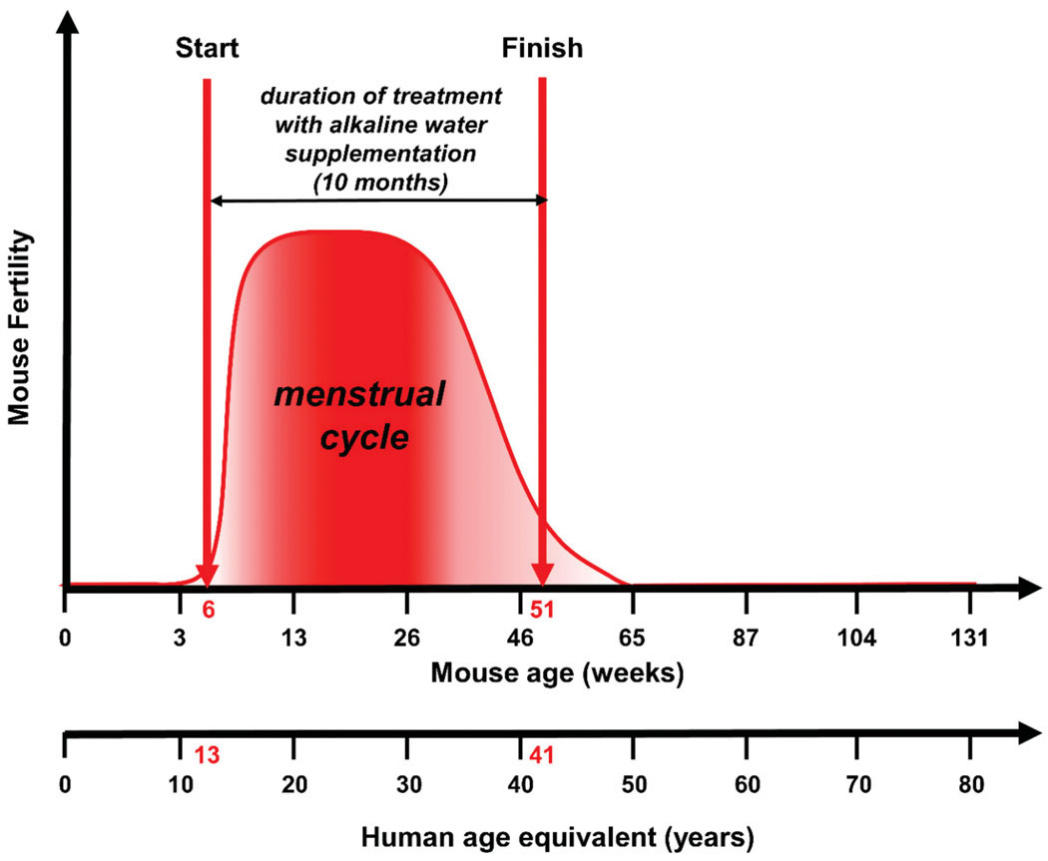
Experimental study design. Evaluation of the antiaging effects of alkaline water supplementation (AWS) (pH 9.0) on C57BL/6J female mice. The treatment with AWS continued for 10 months, starting from 6 weeks of mice age until 51 weeks of mice age, corresponding to the fertile window of human women (13–41 years old).

The objective of our study was the evaluation of the antiaging effects of AWS on C57BL/6J female mice. We have intentionally started treating mice from 6 weeks of age (corresponding to 13 years of human age) up to 11 months of age (corresponding to 41 years of human age; Figure 1). In comparing mice to humans, we knew that the human cycle begins around the age of 13, ending around the age of 45, with a maximum of fertility between 20 and 38 years^50^; while the C57BL mice fertility starts around the 4–7 weeks of age ending at 12 months of age^51^. Therefore, by analogy we have chosen the time window between 6 weeks and 11 months of female mice’ age in order to evaluate the antiaging effect of AWS during an established period of fertile age of mice fully comparable to the same period of female humans.

### C57BL/6J mice blood samples collection

Blood samples collection from CTR and AWS mice was performed by retro-orbital bleeding (ROB) immediately before the sacrifice. This safe phlebotomy technique allowed to obtain high-quality samples of adequate volume (500 µl/mouse) for further analysis^52^. Blood samples were collected in K3-EDTA-coated collection tubes.

### Mice plasma samples

To obtain plasma samples, EDTA-treated whole blood from both CTR and AWS groups was centrifuged at 400 g for 20 min. Plasma samples (250 µl/mouse) were then collected and immediately analysed or stored at −80 °C until analysis.

### Bone marrow cells recovery from C57BL/6J mice

Immediately after the sacrifice of CTR and AWS mice, bone marrow was obtained from both tibias and femurs of the hind legs of mice^53–56^, and placed in physiological solution (NaCl). Bone marrow was disrupted with the blunt end of a 5-ml syringe plunger. Bone marrow cells were isolated using a Falcon^®^ 100 µm cell strainer (Corning, NY), obtaining a uniform single-cell suspension from bone marrow. The single-cell suspensions were washed twice in PBS and immediately processed for the following analysis.

### Ovarian germ cells recovery from C57BL/6J mice

Immediately after the sacrifice of CTR and AWS mice, ovaries were dissected from reproductive system^53–55^^,^^57^ and placed in physiological solution (NaCl) with 1% of trypsin and 0.1 µM of EDTA. Ovaries were isolated from the remaining reproductive system with a cutter and then disrupted with the blunt end of a 5-ml syringe plunger. Ovarian germ cells were isolated using a Falcon^®^ 100 µm cell strainer (Corning, NY), connective tissue and debris were allowed to settle, obtaining a uniform single-cell suspension from ovarian tissue. The single-cell suspensions were washed twice in PBS and immediately processed for the following analysis

### Detection of telomerase by ELISA assay

Quantitative determination of mouse telomerase concentrations was performed in preparations of the plasma obtained from CTR and AWS mice just before the sacrifice. To this purpose, a colorimetric sandwich-ELISA assay, Mouse TE(telomerase) ELISA Kit (Elabsciences^®^, Houston, TE) was used. The optical density (OD) is measured spectrophotometrically at a wavelength of 450 ± 2 nm.

### Detection of telomeres by PNA kit/FITC for flow cytometry

Detection of telomeres was performed in bone marrow cells and in ovarian germ cells of CTR and AWS mice obtained immediately after the sacrifice. To this purpose, a Telomere PNA Kit/FITC for Flow Cytometry (Dako – Agilent, Santa Clara, CA) was used. The kit allows detection of telomeres in nucleated haematopoietic cells using a fluorescence *in situ* hybridisation and a fluorescein-conjugated peptide nucleic acid (PNA) probe. Results are evaluated by flow cytometry using a light source with excitation at 488 nm.

### Total ROS assay

Analysis of the total ROS levels was performed in preparations of the plasma obtained from CTR and AWS mice just before the sacrifice. For this purpose, a total reactive oxygen species (ROS) Assay Kit 520 nm (ThermoFisher, Waltham, MA) was exploited. Plasma samples were incubated with 100 µL of 1× ROS Assay Stain for 60 min in a 37 °C incubator with 5% CO_2_. The samples were treated with the desired reagents to induce production of ROS and analysed on a microplate reader off the 488 nm (blue laser) in the FITC channel.

### GSH detection and quantification assay

Detection and quantification of total glutathione (GSH) levels was performed in preparations of the plasma obtained from CTR and AWS mice just before the sacrifice. To this purpose, a colorimetric assay, Glutathione Colorimetric Detection Kit (ThermoFisher) was used. In order to obtain plasma samples, EDTA-treated blood from C57BL/6J mice were centrifuged at 400 g for 20 min, plasma was collected and immediately analysed. The samples were incubated for 20 min at room temperature after the addition of the detection reagent and reaction mixture. The optical densities were recorded at 405 nm.

### SOD activity assay

Detection and quantification of superoxide dismutase activity were performed in preparations of the plasma obtained from CTR and AWS mice just before the sacrifice. To this purpose, a colorimetric activity assay, The Superoxide Dismutase Activity kit (ThermoFisher, USA) was used. In order to obtain plasma samples, EDTA-treated blood from C57BL/6J mice were centrifuged at 400 g for 20 min, plasma was collected and immediately analysed. The samples were incubated for 20 min at room temperature after the addition of the substrate and chromogenic detection reagent. The optical densities were recorded at 450 nm.

### Statistical analysis

Results in the text are expressed as means ± standard error (SE), calculated using the GraphPad Prism software (San Diego, CA). The statistical analysis was done with an unpaired *t*-test (Student’s *t*-test). Statistical significance was set at *p* < 0.05.

## Results

The experimental design was set up in order to evaluate the effectiveness of the daily AWS (pH 9.0) on the molecular signature of aging. For this purpose, we used a model of female mouse aging, C57BL/6J, divided into two groups: one supplemented with alkaline water and the other left with the usual tap water. At the end of the experiment (10 months) from all mice the blood, the ovaries and the bone marrow were obtained. The blood was analysed for telomerase activity, ROS, SOD-1 and GSH levels; bone marrow and ovaries for telomeres length and the number of cells, as described in the material and methods section. Each result is detailed in the specific sections below.

### Determination of telomerase plasmatic concentration in C57BL/6J mice after 10 month of treatment with AWS

**Figure 2.**
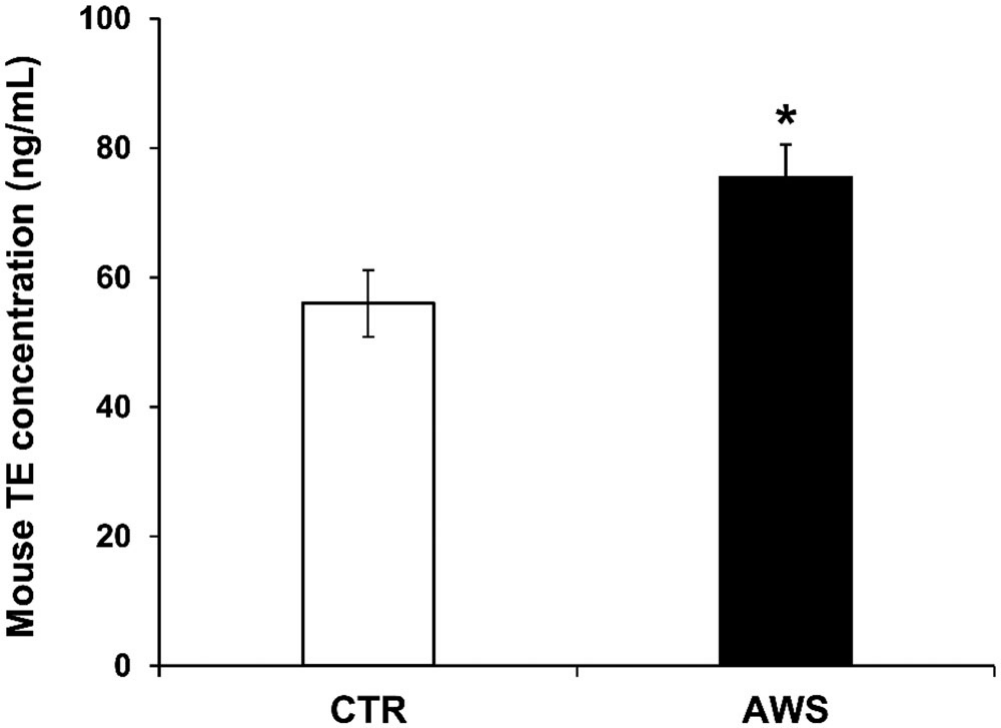
Telomerase (TE) concentration in plasma samples from C57BL/6J female mice treated with alkaline water supplementation (AWS). Quantitative determination of mouse telomerase (TE) concentrations (ng/mL) was performed on the same volume by a colorimetric sandwich-ELISA assay on plasma samples obtained from untreated (CTR group) and treated (AWS group) mice immediately before the sacrifice. CTR group mice received tap water (pH = 7) every day, while AWS group mice were treated orally with AWS (pH = 9.0) for 10 months, every day without interruption, until the sacrifice of the animals. The optical density (OD) is measured to the spectrophotometer at 450 ± 2 nm. Data are normalised on total plasma and expressed as means ± SE. *p < 0.05.

In order to evaluate the possible *in vivo* antiaging effect of AWS, we analysed the telomerase (TE) activity levels in the plasma samples. As shown in Figure 2, we observed an increase of telomerase concentration in plasma of mice daily treated with AWS (AWS group), as compared to the mice drinking untreated water (CTR group). In particular, the amount of TE concentration was 75.5 ± 5.1 ng/mL in AWS group as compared to 56.0 ± 5.2 ng/mL of control group (CTR group) (*p* < 0.05).

This set of results showed that a daily intake of AWS after 10 months of treatment induced a significant increase in the telomerase blood levels (1.4-fold increased enzyme concentrations in AWS group as compared to CTR group).

### Analysis of telomeres length in bone marrow from C57BL/6J mice: AWS group versus CTR group

**Figure 3.**
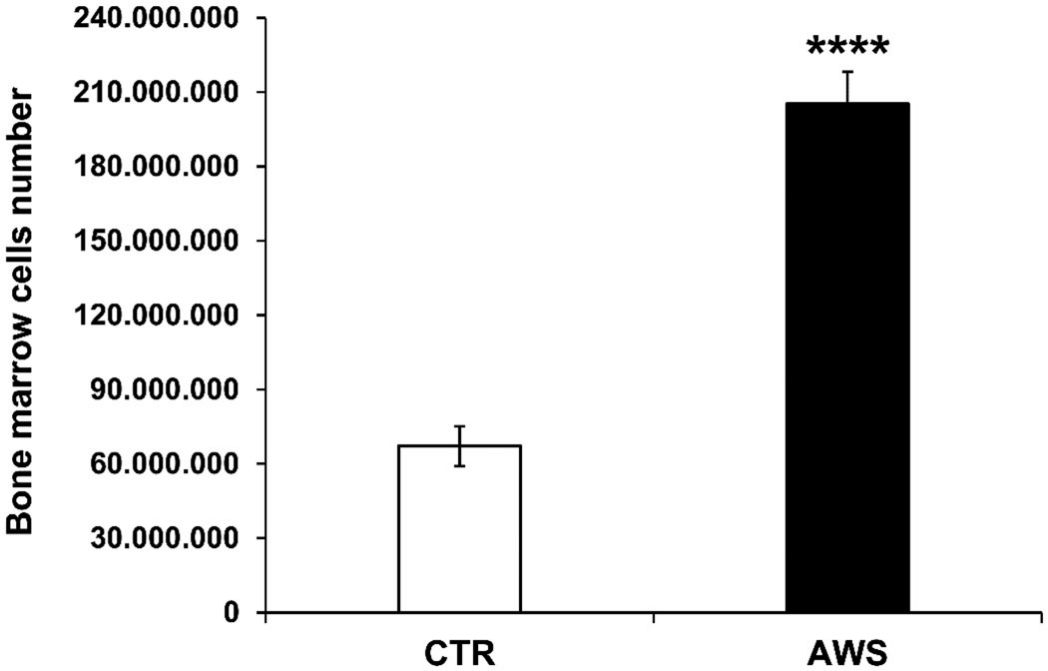
Bone marrow cells number in C57BL/6J female mice. Bone marrow was isolated from both mice tibias and femurs of the hind legs of CTR group and AWS group immediately after the sacrifice, and the resulting bone marrow cells were counted by trypan blue exclusion under optical microscope. ****p < 0.0001.

Bone marrow was isolated from both mice tibias and femurs of the hind legs, and single cell suspensions were obtained, as described^53–56^, from either AWS group or CTR group. Bone marrow cells were counted by trypan blue exclusion under optical microscope. The results are shown in Figure 3; more in details, bone marrow cells were significantly increased, 3-fold higher in AWS group (2.05 × 108 ± 1.29 × 107 cells) as compared to CTR group (6.71 × 107 ± 8.05 × 106 cells) (*p* < 0.0001).

**Figure 4.**
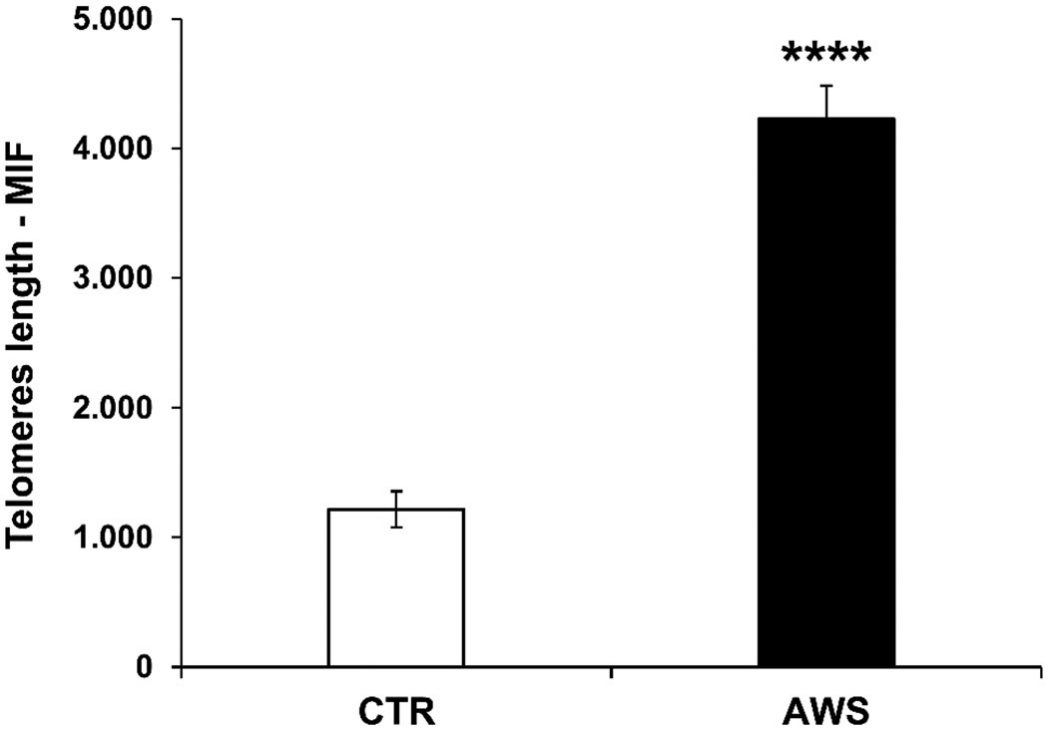
Effect of AWS on length of telomeres in bone marrow cells from C57BL/6J female mice. The analysis of telomeres length mouse was performed on nucleated haematopoietic cells from bone marrow by a fluorescein-conjugated peptide nucleic acid (PNA) probe kit. Cells were obtained from both CTR and AWS groups immediately after the sacrifice. Results are obtained by flow cytometry with excitation at 488 nm. Data are expressed as mean ± SE of mean intensity fluorescence (M.I.F.) normalised on total cells. ****p < 0.0001.

Then, the telomeres length analysis was performed on nucleated haematopoietic cells from bone marrow by a fluorescein-conjugated peptide nucleic acid (PNA) probe kit. The results, obtained by flow cytometry using a light source with excitation at 488 nm, showed that telomeres length was significantly higher (4-fold, *p* < 0.0001) in treated mice with AWS (4226 ± 259 M.I.F.) as compared to control mice (1217 ± 138 M.I.F; Figure 4).

### Analysis of telomeres length in ovarian germ cells from C57BL/6J mice: AWS group versus CTR group

**Figure 5.**
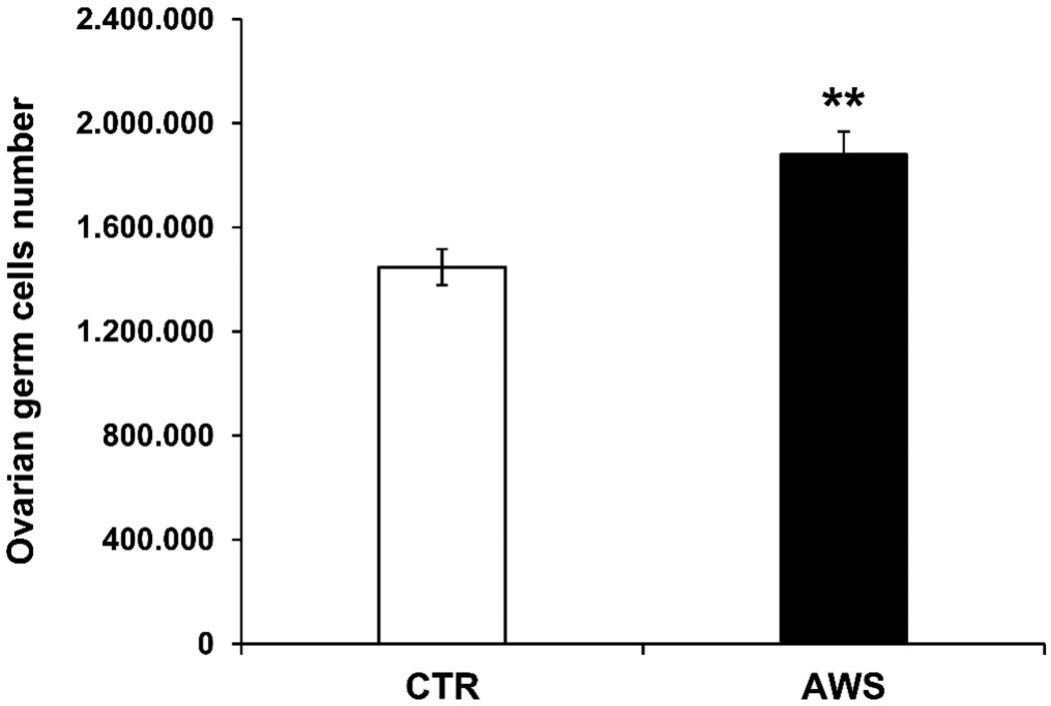
Ovarian germ cells number in C57BL/6J female mice. Ovaries were isolated from both CTR and AWS groups immediately after the sacrifice, and the resulting ovarian germ cells were counted by trypan blue exclusion under optical microscope. **p < 0.005.

We investigated the effect of AWS on telomeres length in ovarian germ cells as well. Single cell suspensions from ovaries of both groups were obtained as detailed in the “Materials and Methods” section and then counted by trypan blue exclusion under the microscope. Comparably to the bone marrow cells, ovarian germ cells were significantly increased (1.3-fold) in AWS group (1.88 × 106 ± 8.85 × 104 cells) as compared to CTR group (1.45 × 106 ± 6.91 × 104 cells; Figure 5; *p* < 0.005).

**Figure 6.**
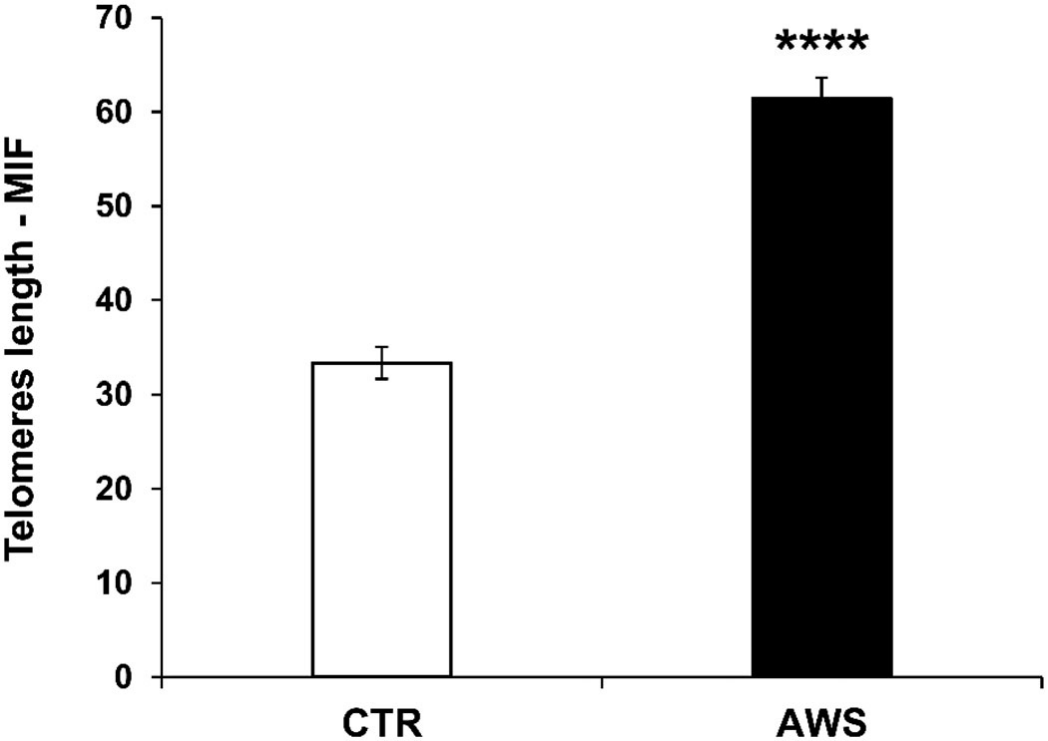
Effect of AWS on length of telomeres in ovarian germ cells from C57BL/6J female mice. The analysis of telomeres length mouse was performed on ovarian germ cells from both CTR and AWS groups immediately after the sacrifice, by using a fluorescein-conjugated peptide nucleic acid (PNA) probe kit. Results are obtained by flow cytometry with excitation at 488 nm. Data are expressed as mean ± SE of mean intensity fluorescence (M.I.F.) normalised on total cells. ****p < 0.001.

When telomeres length was analysed the results showed that telomeres were significantly longer (2-fold, *p* < 0.0001) in ovarian germ cells of AWS mice (61.4 ± 2.2 M.I.F.) as compared to CTR mice (33.3 ± 1.7 M.I.F; Figure 6).

### AWS and plasmatic ROS levels in C57BL/6J mice

**Figure 7.**
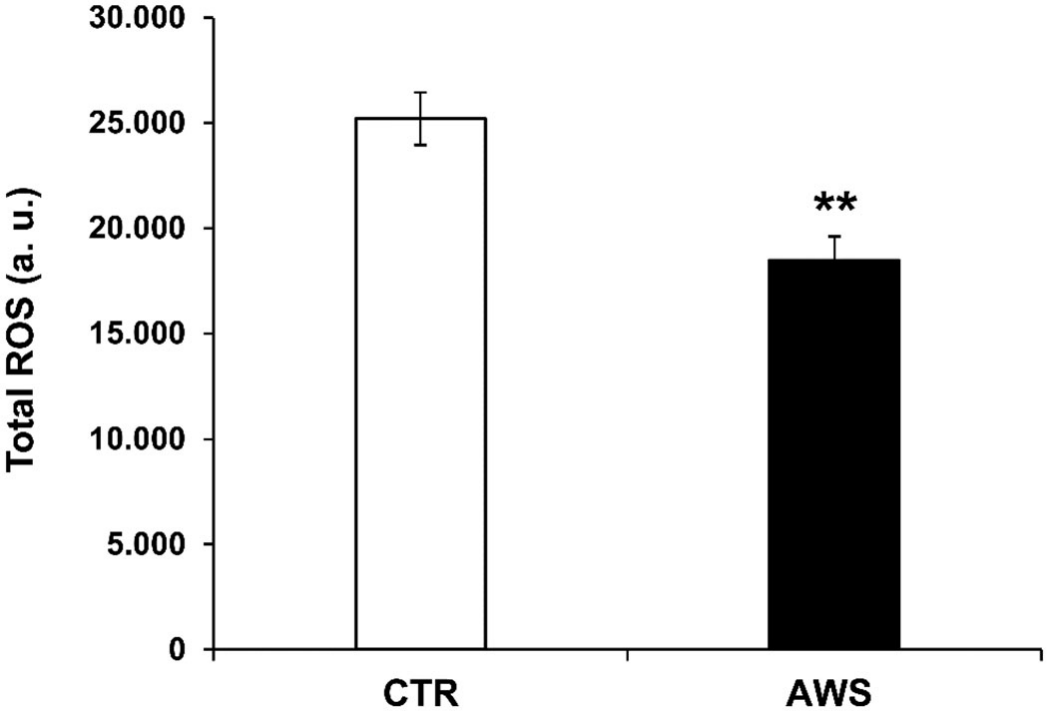
AWS effect on total ROS blood levels in C57BL/6J female mice. Analysis of the total ROS levels (arbitrary units, a.u.) on the plasma samples collected from both CTR and AWS groups immediately before the sacrifice. Analysis of the total ROS levels was performed with a colorimetric assay and measured on a spectrophotometer off the 488 nm (blue laser) in the FITC channel. Data are normalised on total plasma and expressed as means ± SE. **p < 0.005.

In order to evaluate the systemic anti-oxidant response of watering with the supplemented alkaline water, we analysed the redox balance in treated compared to untreated mice. To this purpose, we measured the total ROS levels in plasma samples of both CTR and AW groups, as previously described^34^. The blood was obtained from mice eyes before the sacrifice as previously described^34^. The results showed that AWS induced a highly significant decrease (*p* < 0.005) in the blood ROS levels (18465 ± 1143 a. u.) as compared to CTR group (25201 ± 1255 a. u; Figure 7).

### Plasmatic antioxidant levels (GSH and SOD-1) in C57BL/6J mice treated with AWS

**Figure 8.**
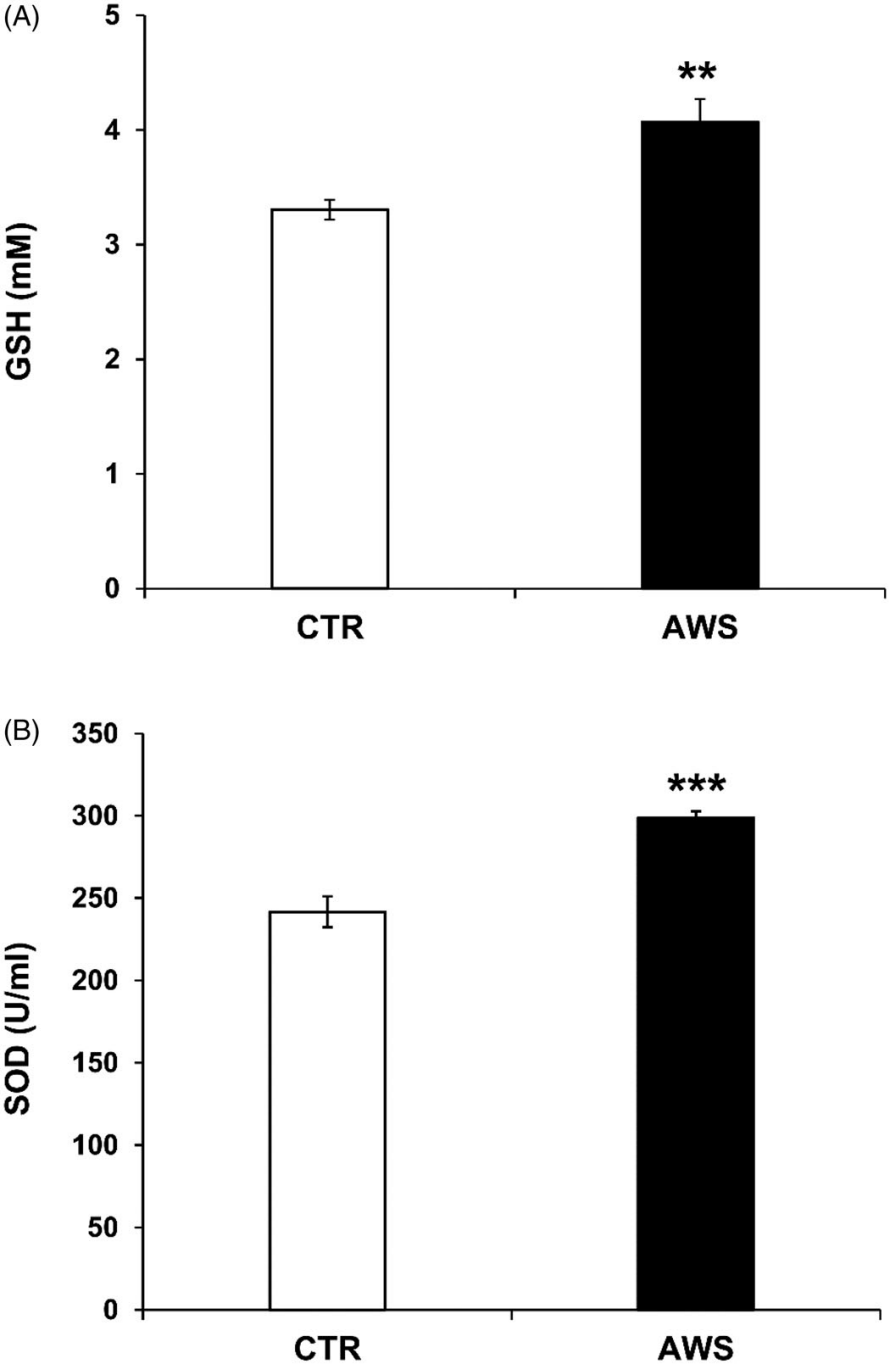
AWS antioxidant effect in C57BL/6J female mice by measuring the plasma antioxidant levels (GSH and SOD-1). Analysis of the total antioxidant activity (glutathione GSH and superoxide dismutase SOD-1) in plasma samples collected from both CTR and AWS groups immediately before the sacrifice. (A) Analysis of the quantification and detection of GSH activity (mM) was performed by a colorimetric activity assay and the concentration was determined by measuring the absorbance at 405 nm. (B) Analysis of the quantification and detection of SOD-1 activity (U/mL) was performed by a colorimetric activity assay and the absorbance was read at 450 nm. Data are normalised on total plasma and expressed as means ± SE. **p < 0.01, ***p < 0.001.

We thus wanted to evaluate the blood levels of natural antioxidants, such as free glutathione (GSH) and enzyme superoxide dismutase-1 (SOD-1) in mice of both AWS and CTR groups. The results clearly showed a significant difference (*p* < 0.01) in GSH levels between the two groups, with the highest levels of GSH in plasma samples of AWS group (4.1 ± 0.2 mM) as compared to CTR group (3.3 ± 0.1 mM; Figure 8(A)).

Similar results were obtained with the SOD-1 blood levels that were again significantly higher in AWS group (298.7 ± 4.1 U/mL, *p* < 0.001) as compared to CTR group (241.6 ± 9.4 U/mL; Figure 8(B)). This set of results showed that the AWS intake lasting 10 months led to increased plasmatic levels of natural antioxidant, with a consistent reduction of ROS levels, witnessing a stable control of the redox balance.

## Discussion

In this study, we evaluated the effect of AWS on the molecular signature of aging, such as telomerase activity and telomeres length, on the systemic redox balance (i.e. the plasmatic levels of ROS, GSH and SOD-1) and the cellularity at the level of the organs under investigation (i.e. bone marrow and ovaries); in female mice treated from 6 weeks to 11 months of age, corresponding to the fertile window of human women (i.e. from 13 to 41 years old). The results have shown that a daily intake of AWS had a clear antiaging effect, as demonstrated by the increase of both telomerase activity and telomeres length, together with a significant reduction of the ROS blood levels with a consistent increase of both GSH and SOD-1 levels. This response to AWS at both systemic and organ levels was also consistent with an increased cellularity in both bone marrow and ovaries. These results supported a critical role of AWS in reducing DNA damage in the whole body, through a clear prevention of the increased oxidation at the cellular and the systemic levels.

Our data were consistent with another study, in which alkaline water had a “sparing effect” on antioxidant enzyme levels in the body. In fact, alkaline water frees up powerful antioxidant enzymes like SOD in a way that they can directly counteract production and tissue accumulation of free radicals into the whole body^58^.

Our results have shown that AWS, supplemented with a series of essential elements, may have a role in improving or at least delaying the aging process. Each of the essential elements may actually contribute to the effects we have shown with the alkaline water. However, in a previous paper the water alkalinisation alone was key in improving the survival of the treated mice as compared to control mice, in a model of long-lived mice^35^. In the 3-year survival study, data were analysed with accelerated failure time (AFT) model showing that a benefit on longevity, in terms of “deceleration aging factor,” was correlated with the consumption of alkaline water alone^35^.

However, some previous papers have shown that water alkalinisation may be useful in both preventing^59^ and treating cancers, while in combination with metronomic chemotherapy^60^, in either a mouse model and in pets with spontaneous tumours, respectively. Inasmuch as we know that cancer suffers of a low pH at the microenvironmental level both papers supported the use of water alkalinisation in implementing existing therapies^25^.

This study is the first showing that watering by using AWS highly improves aging at the molecular level. In fact, we show that AWS induced a 3-fold increase of both telomerase activity into the blood and telomeres length in the bone marrow and ovaries of treated mice. This was consistent with a potent anti-oxidant response at the systemic level, as we previously showed by using a potent anti-oxidant compound, such as fermented papaya (FPP^®^), in treating mice inoculated with a very aggressive melanoma^34^. The ensemble of these results suggests that a daily assumption of AWS may be helpful in both improving aging and reducing the appearance of aging-related tumours in the elderly people, independently from any other factor, including gender. However, we have also shown that in this model of female aging watering with AWS induced an increase of cellularity in the ovaries of treated mice, suggesting an effect that may involve a prolonged fertility, while it has to be investigated with dedicated experiments.

A recent review suggests that a control of the pH balance in our body may well be helpful in reducing the level of bacterial overgrowth in the compartments where there is constitutive cohabitation between bacteria and our epithelial cells (e.g. the gut)^61^. This, in turn suggests that a daily intake of AWS may be key in avoiding bacterial overgrowth during our life. In females, the intake of water and hopefully alkaline water is key inasmuch as water is essential for the full functioning of our natural buffering mechanisms (e.g. HCO3^−^ production). In fact, an outdated paper have shown as acidification underlays osteoporosis^62^, an age-related conditions of women, in turn suggesting that to drink alkaline water is a key way to reduce osteoporosis in women. A more recent clinical study has shown as the daily administration of potassium bicarbonate in post-menopausal women induced bone re-mineralisation^63^, further supporting a critical role of alkaline water for the health of the human beings.

All in all, our results show that a daily assumption of AWS, induce: (i) a systemic antioxidant effect; (ii) an increase in both telomerase activity and telomeres length (i.e. a delay the aging process) and (iii) an increased cellularity in both the bone marrow and the ovaries of the treated mice. These are all phenomena that may well contribute in reducing the appearance of aging-related tumours in the elderly people, prolong the fertility period and hopefully prolong and improve our life as well, simply drinking a substantial amount of alkaline water.
